# A simple contagion process describes spreading of traffic jams in urban networks

**DOI:** 10.1038/s41467-020-15353-2

**Published:** 2020-04-07

**Authors:** Meead Saberi, Homayoun Hamedmoghadam, Mudabber Ashfaq, Seyed Amir Hosseini, Ziyuan Gu, Sajjad Shafiei, Divya J. Nair, Vinayak Dixit, Lauren Gardner, S. Travis Waller, Marta C. González

**Affiliations:** 1 Research Centre for Integrated Transport Innovation (rCITI), School of Civil and Environmental Engineering, University of New South Wales (UNSW), Sydney, NSW 2032 Australia; 2 Department of Civil Engineering, Institute of Transport Studies, Monash University, Melbourne, VIC 3800 Australia; 3 School of Electrical and Computer Engineering, K.N. Toosi University of Technology, Tehran, Iran; 4 Data61, CSIRO, Sydney, 2015 NSW Australia; 5 Department of Civil Engineering, Johns Hopkins University, Baltimore, MD 21218 USA; 6 College of Environmental Design, University of California, Berkeley, CA 94720 USA

**Keywords:** Civil engineering, Complex networks, Statistical physics, Geography

## Abstract

The spread of traffic jams in urban networks has long been viewed as a complex spatio-temporal phenomenon that often requires computationally intensive microscopic models for analysis purposes. In this study, we present a framework to describe the dynamics of congestion propagation and dissipation of traffic in cities using a simple contagion process, inspired by those used to model infectious disease spread in a population. We introduce two macroscopic characteristics for network traffic dynamics, namely congestion propagation rate *β* and congestion dissipation rate *μ*. We describe the dynamics of congestion spread using these new parameters embedded within a system of ordinary differential equations, similar to the well-known susceptible-infected-recovered (SIR) model. The proposed contagion-based dynamics are verified through an empirical multi-city analysis, and can be used to monitor, predict and control the fraction of congested links in the network over time.

## Abstract (web summary)

Predicting and controlling traffic congestion propagation is an ongoing challenge in most urban settings. Here, Seberi et al. apply a contagion model describing epidemic spread in population to model traffic jams, and verify its validity using large-scale data from six different cities around the world.

## Introduction

Traffic congestion in cities, also known as traffic jams, propagate over time and space. Existing approaches to model city traffic often rely on microscopic models with high computational burden as well as excessive parameterization required for calibration^1–3^. Further, the lack of available transportation network data in many countries, especially those that are less economically developed, poses a challenge for traffic modelers. However, the fast-paced development and deployment of mobile sensors offers the opportunity to generate continuous spatial data, which further enables the estimation of road traffic conditions in real time and at the macroscopic level.

Numerous studies have explored different macroscopic approaches to model the spread of traffic jams in cities^4–9^, including through the lens of percolation theory^10,11^, machine-learning methods^12^, and queuing theory^13,14^. However, machine-learning models^12^ and models based on queuing theory^13,14^ are unable to capture and quantitatively describe the propagation and dissipation patterns of congestion over time and space. They are instead formulated based on input (arrival) and output (departure) rates to estimate the number of vehicles (i.e., accumulation) in an area, similar to conservation-based models using Macroscopic or Network Fundamental Diagram (MFD or NFD)^4^. In this work, we show that traffic systems exhibit underlying spreading dynamics similar to those observed and applied in other network systems, for example, social networks, population contact networks, or technological networks. Specifically, we propose and empirically demonstrate that traffic congestion in urban networks can be characterized using a simple contagion process, similar to the well-known susceptible-infected-recovered (SIR) model used to describe spread of infectious diseases in a population, wherein traffic spreads and recovers throughout the network over time. A previous study by Wu et al.^15^ conjectured that traffic congestion spread can be described with a SIR model, but provided no empirical evidence. To the best of the authors’ knowledge, characterizing and modeling congestion propagation and dissipation as a network spreading phenomenon has never been empirically validated.

Urban traffic often exhibits high spatial correlation in which links adjacent to a congested link are more likely to become congested. Additionally, there is a strong temporal correlation in urban congestion, which is known to be driven by the time-dependent profile of travel demand. The spread of congestion at the link level is well theorized and understood with queuing and kinematic wave theories^16–18^. However, our understanding of congestion propagation dynamics at the network level is still incomplete. Queue spillbacks in networks are shown to be sensitive to link capacities^19^, which could remain stable in both under- and over-saturated conditions. Congestion also exhibits fragmentation during recovery^20^ leading to greater spatial heterogeneity and thus results in a drop of network production^21–23^. Propagation and dissipation of gridlocks can be characterized by the number of congested links or the length of congestion in the network^23^ with propagation occurring often at a much higher rate than dissipation. Also, studies have shown that the ratio of road supply to travel demand could explain the percentage of time lost in congestion as an aggregate measure^2,22^.

Unlike individual link traffic shockwaves in a two-dimensional time-space diagram, which are categorized as forward or backward moving, network traffic jams evolve in multiple directions over space. Therefore, we propose that a network’s propagation and dissipation can be characterized by two average rates, namely, the congestion propagation rate *β* and the congestion recovery rate *μ*, which together can predict the number of congested links in the network over time. These two macroscopic characteristics are critical in modeling congestion propagation and dissipation as a simple contagion process^23^.

Despite the complex human behavior-driven nature of traffic, we demonstrate that urban network traffic congestion follows a surprisingly similar spreading pattern as in other systems, including the spread of infectious disease in a population or diffusion of ideas in a social network, and can be described using a similar parsimonious theoretical network framework. Specifically, we model the spread of congestion in urban networks by adapting a classical epidemic model to include a propagation and dissipation mechanism dependent on time-varying travel demand and consistent with fundamentals of network traffic flow theory. We illustrate the model to be predictive, and validate the framework using empirical and simulation-based numerical experiments. The proposed model can be used for adaptive and predictive control of congestion in an urban network. By monitoring the network in real time and observing the number of congested links, the model can be applied to develop optimal control strategies with different objectives, such as minimizing the total duration of congestion, minimizing the total number of congested links, and minimizing the recovery time. Similar to other macroscopic model-based control applications^24–26^, we can employ the proposed SIR-based model to improve the traffic network performance by controlling and optimizing the network input through optimal metering of traffic flow or by increasing the network recovery rate through improved signal timing, bottleneck removal, and capacity expansion.

## Results

### Identifying congested links

We use empirical data from Google that contains estimated time-dependent traffic speeds on every link in the road network across six different cities in the world, namely Chicago, London, Paris, Sydney, Melbourne, and Montreal (see Methods). We also use simulated data from a calibrated and validated mesoscopic dynamic traffic assignment model of Melbourne (see Methods)^1^. Using both empirical and simulation data, we demonstrate that the proposed modeling framework can successfully describe the dynamics of congestion propagation and dissipation in urban networks.

**Fig. 1:**
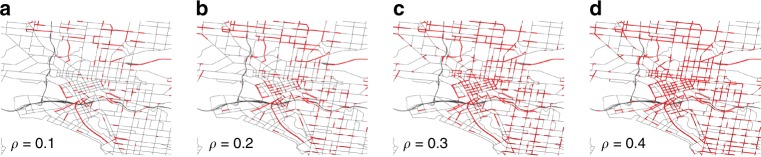
Spatial distribution of congestion in the Melbourne network. At a given time *t* = 8:30 a.m. maps indicate the congested links (coded red) on the network when **a** *ρ* = 0.1, **b** *ρ* = 0.2, **c** *ρ* = 0.3, and **d** *ρ* = 0.4.

In the proposed network-theoretic framework, nodes represent the intersections (controlled and uncontrolled) and links represent the physical roads between any two intersections. For each link *i* in the network, we have the time-dependent speed *v*_*i*_(*t*). We represent the maximum speed on the link as $v_i^{{\mathrm{max}}}$. To reveal how congestion propagates and dissipates in a network, we define

1 $$\lambda _i(t) = \frac{{v_i(t)}}{{v_i^{{\mathrm{max}}}}},$$

where *λ*_*i*_(*t*) is the ratio of link speed *v*_*i*_(*t*) over link maximum speed $v_i^{{\mathrm{max}}}$. We then classify each link as in either a congested *s*_*i*_ = 1 or uncongested *s*_*i*_ = 0 state (also known as free flow) using a threshold *ρ* as below

2 $$s_i(t) = \left\{ {\begin{array}{*{20}{c}} {1,} & {\lambda_i(t)\;<\;\rho }, \\ {0,} & {\lambda_i(t)\ge\rho }, \end{array}} \right.$$

where *ρ* is a pre-specified threshold that represents different congestion levels^11^. Figure 1 illustrates the identified congested network for different values of *ρ* at a given *t* using data from the simulation-based dynamic traffic assignment model of Melbourne. The size of the congested network grows as *ρ* increases. Alternatively, one can also construct the congested network using traffic density measurements or any other classification method.

### Modeling contagion dynamics of network traffic jams

Consider a network with *N* directed links. At time *t* = 0 every link in the network is in the free flow or uncongested regime, *F* (0) = *N* and no link is congested *C*(0) = 0. Let *E* be the set of links, *j*(*j* ∈ *E*) of length *l*_*j*_ on which congestion later forms and propagates throughout the network. *F*(*t*) represents the number of links that are at free flow regime at time *t* and *C*(*t*) represents the number of congested links at time *t* based on the specified *ρ*. For a directed link in the road network with on average *k* “effective contacts” with other links pointing at its upstream node, *β* is the rate of congestion propagating to an upstream free flow link in unit time, and is *F*(*t*)/*N* is the probability of a congested link being directly connected downstream of a free flow link. Under the assumption of homogeneous mixing, which refers to an assumption that each link in the network has the same probability of contact with a congested link; a congested link connects to an average of *kF*(*t*)/*N* free flow links in a unit time. Although the assumption of homogeneous mixing is simplistic, it makes the analysis tractable while shown to be predictive at the macroscopic scale. See Supplementary Note 1 for the fundamentals of congestion propagation in simplified traffic systems. See Supplementary Note 2 for the extension of the framework when the homogeneous mixing assumption is relaxed.

Below, we describe the dynamics of congestion propagation with a system of ordinary differential equations (ODEs), which is analogous to the well-known SIR model:

3 $$\frac{{{d}c(t)}}{{{d}t}} = - \mu c\left( t \right) + \beta kc\left( t \right)\left( {1 - r\left( t \right) - c\left( t \right)} \right),$$

4 $$\frac{{{d}r(t)}}{{{d}t}} = \mu c\left( t \right),$$

5 $$\frac{{{d}f(t)}}{{{d}t}} = - \beta kc\left( t \right)\left( {1 - r\left( t \right) - c\left( t \right)} \right),$$

where *c*(*t*) represents the fraction of congested links in the network, *f*(*t*) is the fraction of free flow links, and *r*(*t*) is the fraction of recovered links. Equation 3 describes the rate in which *c*(*t*) changes over time given a propagation rate *β* and recovery rate *μ* considering that a fraction of congested links will eventually recover as demand for travel diminishes. Equation 4 expresses the rate at which congested links recover given recovery rate *μ*. Equation 5 represents how the fraction of free flow links *f*(*t*) in the network changes over time given *c*(*t*) and *r* (*t*). Note that *c*(*t*) + *r* (*t*) + *f*(*t*) = 1, where *f*(*t*) represents links that have remained in a free flow state from *t* = 0. Also, *kβ*/*μ* represents the average number of newly congested links each already congested link potentially creates, in a fully freely flowing road network. In epidemic modeling, this is often denoted by *R*_0_ and called the “basic reproductive number”. The higher is *R*_0_, the faster congestion spreads throughout the network. If *R*_0_ ≤ 1, congestion will not spread in the network and remains a non-persistent local phenomenon. *R*_0_ in an urban network can be estimated at the onset of congestion, before congestion starts propagating in the network. Congestion propagation in an urban network often occurs over a few hours from the onset of congestion and recovers within a few hours after the peak point. If *R*_0_ is known, as soon as congestion forms and starts to propagate, the proposed SIR model can be used to predict when congestion will peak and how long it takes to recover, which can be used to optimize the implementation of various traffic management and control strategies.

**Fig. 2:**
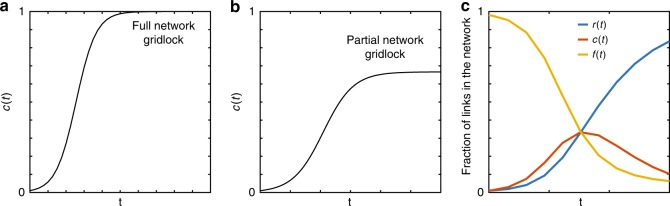
Describing temporal evolution of congestion. Two-state model of congestion propagation in a network: fraction of congested links *c*(*t*) vs. time resulting in **a** full network gridlock described by a variation of the SI model and **b** partial network gridlock described by a variation of the SIS model. **c** Three-state model of congestion propagation and dissipation in a network, analogous to the SIR model subject to a time-varying loading–unloading demand profile.

The formulated model simultaneously describes the dynamics of congestion propagation as well as congestion dissipation or recovery in a network, given estimated parameters *β* and *μ*, which are dependent on a definite time-dependent travel demand profile as in real-world networks. Analogous representations to the well-known susceptible-infected (SI) and susceptible-infected-susceptible (SIS) model of epidemics can also be formulated to describe the spread of traffic in a network as a two-state model (see Supplementary Note 1). If a SI model is adopted, the estimated *c*(*t*) describes propagation of congestion in the network as long as demand for travel continues and congestion eventually propagates to the entire network with no recovery leading to a full gridlock, also known as “complete jam”^27,28^ or “collapse of the network”^29^. Here, gridlock is defined as a state of the system under which traffic in the entire network or a portion of the network comes to a complete standstill with zero (or minimal) flow^20^ (see Fig. 2a). If an SIS model is adopted, the congestion propagation dynamics are described such that congestion continues to grow but does not propagate to the entire network and remains invariant after some time leading to a partial network gridlock (see Fig. 2b). While from a theoretical perspective, both SI and SIS models could also be adopted to describe congestion propagation in a network, they fall short of realism. In a partial network gridlock, the number of congested links can grow or shrink depending on whether the gridlock is propagating or resolving itself (dissipating) as what often happens in real-world networks. Therefore, we expect that the SIR model, as a three-state model, provides a more realistic representation as will be empirically demonstrated in the next section (see Fig. 2c).

### Empirical evidence

**Fig. 3:**
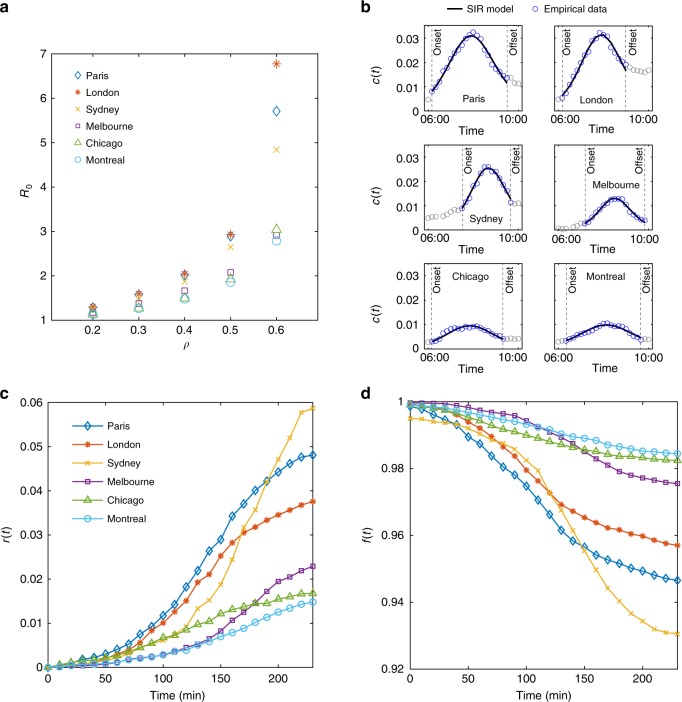
Empirical evidence on the congestion propagation and dissipation dynamics from six different cities. **a** Relationship between *R*_0_ and *ρ*. For smaller values of *ρ*, there is a consistency in the observed dynamics across different cities despite their differences in network structure, demand, and traffic patterns.*R*_0_ remains roughly invariant for the same value of *ρ* for smaller *ρ* values, suggesting the existence of a universal measure for congestion propagation; **b** proportion of congested links in the network over time *c*(*t*) for each city from 6:00 to 10:00 a.m. when *ρ* = 0.2. Congestion onset and offset is marked with dashed lines. Note that the *y*-axis has a fixed range [0, 0.035] and a subset of data between the congestion onset and offset times are utilized to fit the SIR model; **c** evolution of fraction of recovered links *r*(*t*) in the network over time; **d** evolution of the fraction of free flow links *f*(*t*) in the network over time.

We apply the proposed contagion-based model to empirical data collected from six different large metropolitan cities around the world (see Fig. 3a–d). We explore changes in the fraction of congested links *c*(*t*) in the selected networks. The proposed dynamics in Eq. 3–5 are fit to traffic data between the onset and offset of congestion (Fig. 3b) to estimate the propagation rate *β* and the recovery rate *μ* using an ordinary least-square (OLS) method with a pattern search algorithm (see Methods) for different values of *ρ* as in Eq. 2 and assuming an average *k* = 3 for the studied networks based on the distributions of the node degree.

The applied simple contagion process successfully describes the congestion spreading patterns in different cities at a macroscopic level. While the selected cities have significantly different topology and travel demand patterns, their estimated *R*_0_ values are almost the same for *ρ* = 0.2 and only slightly vary for *ρ* = 0.3 (see Fig. 3a). This suggests existence of a universal measure represented by *R*_0_ for the spread of congestion in urban network similar to what is usually observed for infectious diseases. Note that *c*_max_ and the time where *c*_max_ occurs are different between cities. For larger *ρ* values, the observed difference between the estimated *R*_0_ grows, which is mainly due to the loose definition of congestion when *ρ* is large. Therefore, we expect to see divergence between cities as *ρ* increases. The fraction of recovered and free flow links in the studied networks is also illustrated in Fig. 3c, d, and shown to be consistent with the expected outcomes of the theoretical SIR model.

### Revealing the underlying dynamics with simulation

**Fig. 4:**
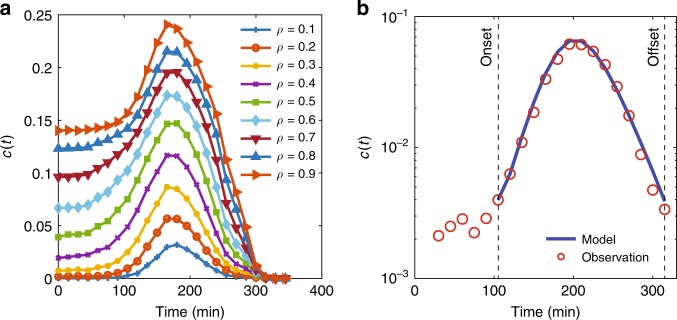
Propagation and dissipation of congestion in Melbourne network. **a** Evolution of fraction of congested links in the network over time for various *ρ* subject to realistic and calibrated 4-h demand followed by a 4-h recovery period with zero demand starting at *t* = 240 min. **b** Fitted SIR model to dynamics of the system with estimated parameters *β* = 0.0577 and *μ* = 0.0812 when *ρ* = 0.2 and *k* = 2.12. Data are obtained from the simulation-based DTA model. The vertical axis associated with *c*(*t*) is scaled logarithmically.

We now explore changes in the fraction of congested links *c*(*t*) in the network, given the traffic data obtained from the simulation-based dynamic traffic model of Melbourne (Supplementary Note 3) and the identified congested links. See Supplementary Note 4 for a comparison between empirical and simulation-based data from the Melbourne network. Figure 4a shows the evolution of *c*(*t*) over time for different values of *ρ* using simulated data with a calibrated travel demand profile for the morning peak period 6:00–10:00 a.m., followed by a 4-h recovery period with zero demand. Introducing zero demand after a complete network loading is a common approach in network traffic analysis^2,21^, which allows the network to go under a complete recovery. Many of the interesting properties of network traffic flow can only be observed during a full recovery such as the formation of hysteresis in the network flow-density relationship.

The simulated congestion propagation and dissipation patterns follow the commonly observed spreading patterns in epidemics in which the propagation follows an initial exponential growth regime, followed by a sum of multiple exponential processes during recovery. Smaller values of *ρ* are used as they better reflect congestion formation compared to larger values of *ρ*. For example, when *ρ* = 0.1 is being used, the fraction of congested links in the network is almost zero for the first hour of the simulation as congestion has not yet formed in any link across the network. However, when *ρ* = 0.9 is used, nearly 15% of the links in the network are considered congested at the beginning of the simulation. The adopted and formulated SIR model expressed in Eq. 3–5 successfully describes the evolution of *c*(*t*) over time as shown in Fig. 4b. The model is applied to traffic data between the congestion onset and offset times at which the traffic jam starts propagating and almost finishes dissipating, respectively.

**Fig. 5:**
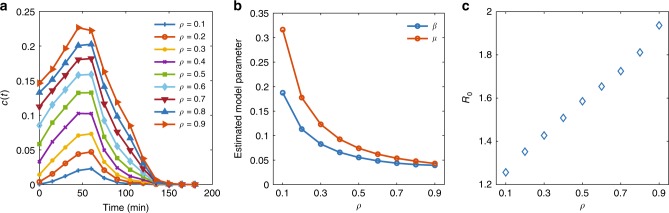
Spread of traffic congestion in Melbourne network. Propagation and dissipation of congestion in the Melbourne network subject to 1-h peak demand, followed by a recovery period with zero demand starting at *t* = 60 min: **a** evolution of fraction of congested links in the network over time for different values of *ρ*, **b** estimated model parameters *β* and *μ* for various *ρ*, **c** sensitivity of *R*_0_ to *ρ*. Here, *R*_0_ > 1 suggests that congestion is spreading for every value of *ρ* > 0.

To further reveal the congestion spreading patterns, we also conduct a simulation with 1 h of peak demand loading followed by several hours of recovery with zero demand, consistent with an analysis previously reported by Olmos et al.^2^. Here we focus on a target group of simulated vehicles that enter the network within the peak hour (8:00–9:00 a.m.), specified as the hour immediately before the peak point on the demand profile. See Fig. 5a in which congestion propagation in the network can be approximated by an initial growth followed by a decay. Figure 5b, c illustrates the changes in the estimated parameters *β*, *μ*, and *R*_0_ for a range of *ρ* values. It is counter-intuitive that when *ρ* increases, the rates of congestion propagation and dissipation both decrease exponentially. However, *R*_0_ increases when *ρ* increases as expected. In fact, propagation and dissipation rates here must be interpreted relatively. Therefore, their individual values may not provide an absolute indication of the extent that congestion is spreading. Instead, it is the ratio of *β* over *μ*, or more accurately *R*_0_, that has a physical meaning. This is analogous to queuing theory in which the size of a queue depends on the difference between the arrival and departure curves rather than the individual arrival and departure rates. Here, *R*_0_ can also be seen as a representation of the rate in which the network shockwave evolves. Further, *R*_0_ follows an interesting linear relationship with *ρ*, as shown in Fig. 5c.

### Local congestion propagation dynamics

**Fig. 6:**
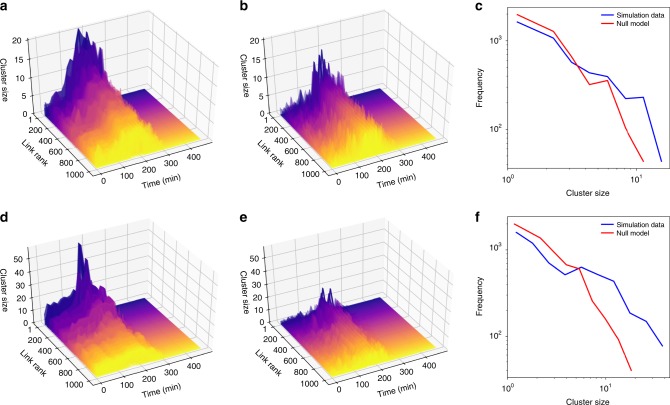
Evolution of the congested upstream clusters associated with congested links. Here, **a** and **d** show the change over time in the size of the congested clusters associated with links in Melbourne’s traffic network, while **b** and **e** depict the same results for the null model generated independently for each time step by drawing random *λ*_*i*_(*t*)’s for each link *i* from the same distribution as in the simulated data; links are sorted according to the maximum size of their congested upstream cluster and the results only show the top 1000 links. The distribution of the size of the congested upstream clusters for the Melbourne traffic simulation model and its null model counterpart at *t* = 180 is compared for **c** *ρ* = 0.5 and **f** *ρ* = 0.7. Larger congested upstream clusters are observed more frequently in the Melbourne traffic network model compared to the null model generated with the same structure and the same distribution of the link relative velocity.

In order to further investigate the spreading properties of congestion, we demonstrate the propagation of congestion from congested links to their upstream links in the network using simulated traffic data. Specifically, we reveal the formation of congested clusters at the upstream of congested links. For any directed link *i* connecting its source node to its target node, let us define the corresponding congested upstream cluster as the subset of nodes on the network that can reach link *i*ʼs source node via at least one directed path entirely consisted of congested links; the cluster includes the source node. For a fixed *ρ*, we individually consider each congested link, and calculate the size of its congested upstream cluster at different points in time. For each time step *t*, we generate a null model by randomly drawing *λ*_*i*_(*t*) of each link *i* from the same distribution of the link relative speeds as in the simulated data at the same time step. To measure the significance of the impact of congested links on their neighborhood, we compare the size of the congested upstream cluster associated with each link on the simulated network with that of the generated null model, over time (see Fig. 6). Results indicate the spatial distribution of link *λ*’s over the simulated network is significantly different from the null model. In particular, there are significantly more congested upstream clusters of larger size in the simulated traffic network compared to the null model. In other words, in the traffic network, links with lower *λ*_*i*_(*t*) tend to emerge inside small- to medium-sized clusters, while this phenomenon is not observed in the null model. Furthermore, the size of the congested upstream clusters in the simulated network changes over time. It starts from low values around *t* = 0, and gradually increases until approximately *t* = 200 min when it peaks for most of the links, and then decreases toward zero until around *t* = 400 min, when the network is empty. However, for the null model the size of the clusters keeps fluctuating over time with large variations, and the size of the congested upstream clusters only reaches half of what is observed in the simulated Melbourne traffic network. Consistent with the physics of traffic flow and kinematic wave theory, this verifies the hypothesis that congestion at the link level spreads over the network via upstream links. We illustrate the difference in the distribution of the congested upstream cluster size in the simulation network to that of the null model at time *t* = 180 min as an example (see Fig. 6c for *ρ* = 0.5 and Fig. 6f for *ρ* = 0.7). The observed difference between the distributions of the cluster size in the simulated data versus the null case confirms that congestion follows a non-random spatial spreading pattern.

### Connection with travel demand

**Fig. 7:**
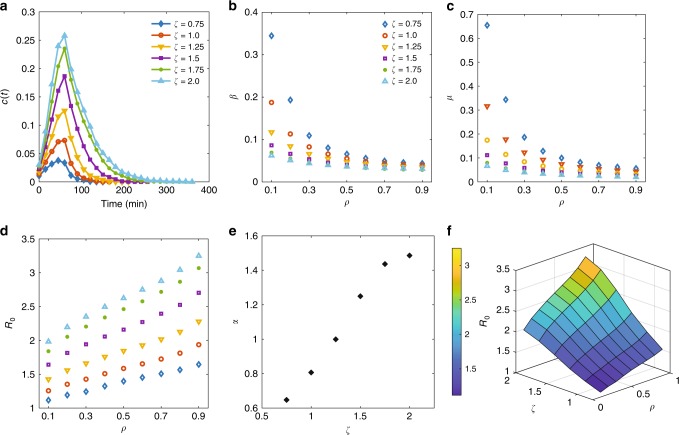
Effect of travel demand on congestion dynamics. Propagation and dissipation of congestion in the Melbourne network subject to 1-h demand followed by a recovery period: **a** evolution of fraction of congested links in the network over time for various demand levels given *ρ* = 0.3; **b** and **c** estimated model parameters *β* and *μ* for various *ρ* and demand levels; **d** *R*_0_ as a function of *ρ* for various demand levels; **e** sensitivity of *α* to *ζ*. Here, *α* represents the slope of a linear fit to the data shown in **d**. **f** Three-dimensional illustration of the relationship between *R*_0_, *ρ*, and *ζ*.

Here, we examine the impact of changing demand on the propagation and dissipation dynamics of congestion. For the same network with 1 h of loading followed by 4-h recovery, we have conducted multiple simulation runs with a scaling factor of *ζ* = 0.75, 1.0, 1.25, 1.5, 1.75, and 2.0 to decrease or increase demand while keeping the origin–destination demand patterns the same. We then solve the proposed ODE model and estimate the model parameters *β* and *μ* as described in Supplementary Note 5 and Methods section. When demand increases, the fraction of congested links also increases for the same network, and as a result, complete recovery of congestion takes longer while the start of the recovery phase remains the same. This is reflected in the reduction of *μ* in response to the increase in demand for any given *ρ* (see Fig. 7a for the evolution of *c*(*t*) over time for various demand levels). Counter-intuitively, increasing demand also results in reduction of *β* for a given *ρ* (see Fig. 7b, c). This should not be interpreted as a slower propagation of congestion. In fact, *β* is not independent of *μ*. The two macroscopic characteristics vary interdependently, so *c*(*t*) reaches its peak value at *t* = 75 min according to the demand profile. However, *R*_0_ increases when demand increases for any given *ρ* with a clear indication that the size of the network shockwave will grow larger and it takes longer to recover. *R*_0_ has a linear relationship with *ρ* in which the slope also follows an almost linear relationship with demand (see Fig. 7d, e). Results suggest that there is also a three-dimensional relationship between *R*_0_, *ρ*, and demand as illustrated in Fig. 7f, in which for smaller values of *ρ*, the relationship between *R*_0_, and demand is almost linear. The relationship with demand is critical for applying the proposed model to travel demand management and traffic control in real world. The proposed model can be used for perimeter control of a sub-network within a larger network, in which the relationship with demand guides which *R*_0_ should be used given a fixed *ρ* value, and vice versa. When *R*_0_ is known, the proposed model can then predict when the congestion will peak and when it fully recovers. Identifying the time of the congestion peak and recovery duration can be used to identify the time-optimal traffic control strategies (see Supplementary Note 6). What is missing here is the observed relationship with demand from empirical data. Travel demand is difficult to observe in real world. Therefore, our analysis here remains limited to the simulation environment. Passive mobile phone use data could potentially be used to obtain travel demand over time and relate with congestion propagation and dissipation in the network, which remains an interesting direction for future research.

## Discussion

This study has shown that propagation and dissipation of traffic jams in cities can be described by a simple contagion process, which is formulated as a system of ODEs, analogous to the well-known SIR model, dependent on two macroscopic network traffic flow characteristics, namely congestion propagation and congestion recovery rate. The paper has shown both empirically and simulation based that congestion propagation is indeed a spreading phenomenon. The extent to which congestion builds up in a network and how fast it recovers are shown to be dependent on the ratio of the propagation over recovery rates represented by the basic reproductive number *R*_0_. Using data from a simulation-based dynamic traffic assignment model of Melbourne, we showed that time-dependent travel demand profile has, as expected, an impact on the dynamics of congestion propagation and dissipation in a network. In fact, *R*_0_ increases when demand increases for any given *ρ* with a clear indication that the size of the traffic jam will grow larger and it takes longer to recover in response to travel demand. Moreover, empirical data from six different cities are analyzed to verify the validity of the proposed contagion-based model; see also Supplementary Note 7 on the case of a very small urban road network. We observed that different cities surprisingly exhibit very similar *R*_0_ for small *ρ* values, indicating that at the macroscopic level, they tend to have consistent congestion propagation dynamics. A limitation of this study is that in the classical SIR model when an infected individual recovers, he/she will not be infected again (or removed). This clearly does not apply directly to traffic networks in which a link may recover and become congested again after a short period. However, the assumption may still hold if the temporal aggregation of analysis is large enough to prevent links to go under multiple cycles of loading and recovery in a short sequence of time steps. Given the unimodality of the travel demand profile during a peak period (morning or afternoon), it may not be unreasonable to assume, at a macroscopic level, that when a link recovers from congestion, it will not become congested again until the next peak period. Nevertheless, relaxation of this assumption and introduction of other traffic states considering multiple cycles of loading and recovery should be an item of research priority in the future.

## Methods

### Traffic simulation model

Simulation of the Melbourne network is conducted in AIMSUN, a commercially available traffic simulation software. The model is a mesoscopic simulation-based dynamic traffic assignment, which has been calibrated and validated for the morning peak period 6:00–10:00 a.m. A large number of input (demand and supply) parameters need to be calibrated before the simulation outcomes are used. Details of the calibration and validation process can be found in ref. ^1^ and Supplementary Note 3.

### Google traffic data

Google traffic data on 27 June 2018 are acquired and used, which consist of speeds in the unit of km/h for each link in the network with a unique identifier. The time frame for congestion modeling varies from city to city, which is determined based on the profile of the fraction of congested links, so as to get a fitted model between the onset and offset times of congestion. Nevertheless, the total time window remains the same as in our simulation (i.e., 6:00–10:00 a.m.). Links with missing data for the entire day are discarded in our analysis, but the number of such links is negligible and does not affect the results. A direction for future research is to extend the analysis to multiple days to study the day-to-day spreading dynamics of network traffic.

Google traffic data record the speeds of road sections at continuous time intervals. The data were estimated using floating GPS points of mobile users once they used Google services such as Google Maps. The sampling interval of the data is 10 min, which provides an appropriate temporal resolution for our analysis. Cities that are selected include Melbourne, Sydney, London, Paris, Chicago, and Montreal, which all have a complex urban transport network with a dense population, thereby ensuring the availability of GPS data in large volumes. Note that the reliability of estimations is directly proportional to the amount of data. The calculation of $v_i^{{\mathrm{max}}}$ takes into account the maximum speed observed on link *i* in a cycle of one day (i.e., 24 h). Using this measure along with *v*_*i*_(*t*), the ratio *λ*_*i*_(*t*) is calculated for the entire network. To be consistent, only the morning peak of each city is considered in our analysis, which all lies in the time period between 6:00 and 10:00 a.m., although being subject to some differences from city to city.

### Parameter estimation with global pattern search

To estimate the parameters of the proposed model, we follow a similar approach presented in ref. ^30^. The estimation is performed using an OLS method with a pattern search algorithm. The estimation is formulated as a minimization problem in which the pattern search seeks to find the model parameters that minimizes the root mean-squared error (RMSE) calculated as the deviation between the observed and modeled *c*(*t*) over the study time period. We have applied the global pattern search algorithm as a derivative-free global optimization method in which the modeled curves for *c*(*t*) are fit to the traffic data with the objective function to minimize the RMSE. The pattern search algorithm falls under the general category of global optimization methods in which an initial mesh is first specified in the solution space given an initial guessed solution. The algorithm computes the objective function value at each mesh point until it finds a point whose value is smaller than the objective function value at the initial solution point. The algorithm then updates the mesh size and re-computes the objective function value at each mesh point. This will iteratively continue until one of the stopping criteria is met, such as the mesh size getting smaller than a mesh tolerance threshold or if the maximum number of iterations is reached. For details about the pattern search algorithm, see ref. ^31^. Also see Supplementary Table 1 in Supplementary Note 5 for the estimated model parameters and associated RMSE for different values of *ρ*.

## Supplementary information

Supplementary Information

## Data Availability

For contractual and privacy reasons, we cannot make the empirical data from Google available. However, all data from the simulation-based dynamic traffic assignment model that are needed to replicate the findings reported in the paper and Supplementary Information are available at https://github.com/meeadsaberi/trafficspreading.

## Code availability

The simulation-based dynamic traffic assignment model of Melbourne is also available as an open access model, which can be downloaded via https://github.com/meeadsaberi/dynamel.
