# Supplementary material for: A simple contagion process describes spreading of traffic jams in urban networks
Source: Nat Commun. 2020 Apr 7;11:1616. doi: 10.1038/s41467-020-15353-2 (PMC7138808; doi:10.1038/s41467-020-15353-2)
Supplement: Supplementary file 1 — Supplementary Information [file 41467_2020_15353_MOESM1_ESM.pdf]

Supplementary Information for

A simple contagion process describes spreading of traffic jams in urban networks

Meead Saberi, Homayoun Hamedmoghadam, Mudabber Ashfaq, Seyed Amir Hosseini, Ziyuan Gu, Sajjad Shafiei, Divya J. Nair, Vinayak Dixit, Lauren Gardner, S. Travis Waller, Marta González

Supplementary Note 1. Fundamentals of congestion propagation from traffic flow theory perspective

The simplest system to study is a single one directional corridor with a set of links E^c, i $(i \in E^c)$ of equal length l. Denote by $F(t)$ the number of links that are at free flow regime at time t and by $C(t)$ the number of congested links at time t. Let's assume flows and densities are related by a triangular fundamental diagram with maximum flow q_c, critical density k_c, free flow speed v_f, and jam density k_j as shown in Supplementary Figure 1. Let ω denotes the shockwave speed representing the transition from jam density to capacity $\omega = q_c/(k_j - k_c)$. Therefore, the time that it takes for congestion to propagate from link i to the upstream link $i + 1$ is

$$\eta = \frac{l}{\omega} = \frac{l(k_j - k_c)}{q_c} \tag{1}$$

If congestion continues to propagate given an indefinite demand and no recovery with a fixed shockwave speed of ω, the number of newly congested links $dC(t)$ in the corridor in a time interval dt can be expressed as

$$\frac{dC(t)}{dt} = \frac{1}{\eta} \tag{2}$$

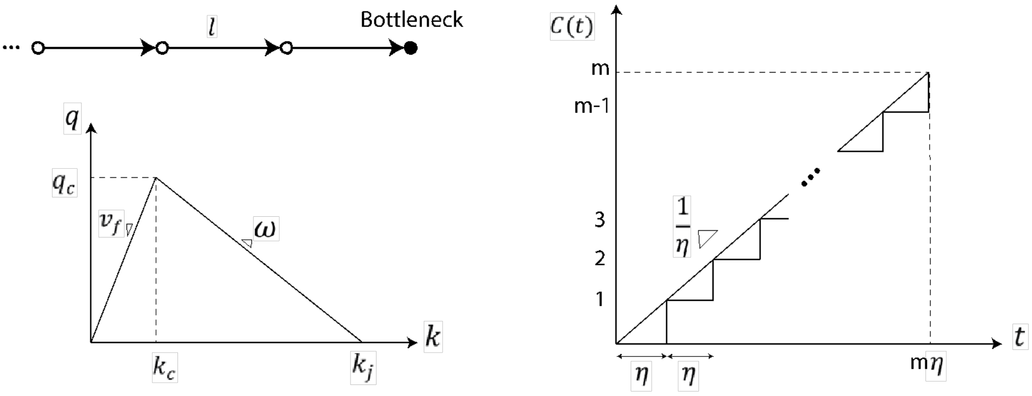

Supplementary Figure 1. A single corridor consisting of m links upstream of a bottleneck with equal length l with an assumed triangular fundamental diagram and a fixed shockwave speed of ω. The number of congested links in this simple system can be described by a linear relationship with a fixed slope of $1/\eta$.

However, if congestion follows a time-dependent demand profile as in real-world networks, the rate that $C(t)$ varies over time will not remain constant. For instance, let us assume a step-wise time-dependent demand profile in which flow begins with q_0 at $t = 0$ and increases to q_c at $t = t_1$ and then decreases back to q_0 at $t = t_2$ as shown in Supplementary Figure 2(a). Therefore, the number of congested links $C(t)$ at $t = t_1$ and $t = t_2$ can be respectively expressed as

$$C(t_1) = \frac{1}{\eta_0} t_1 = \frac{\omega_0}{l} t_1 \quad (3)$$

$$C(t_2) = \frac{1}{\eta}(t_2 - t_1) + C(t_1) = \frac{\omega}{l}(t_2 - t_1) + \frac{\omega_0}{l} t_1 \quad (4)$$

In this case, the number of congested links $dC(t)$ in the corridor in a time interval dt can be described as a piecewise linear function as shown in Supplementary Figure 2(a). This can simply be extended to a case in which the demand profile follows a continuous form $q(t)$. See Fig 2(b) for an illustrative example. Therefore, Supplementary Equation 2 can be re-written as follows which is a continuous function of flow $q(t)$ at any given time t, creating dynamics shockwaves.

$$\frac{dC(t)}{dt} = \frac{1}{\eta(t)} = \frac{q(t)}{l(k_j - \frac{q(t)}{v_f})} \quad (5)$$

This paper extends this simple case to a network and proposed an analytical formulation that describes the congestion spreading phenomenon with a continuous approximation of the rate in which $C(t)$ changes over time. We also argue and demonstrate that the congestion propagation dynamics in a network with a continuous loading-unloading demand profile follows an exponential growth and recovery similar to spread of epidemics in a social network.

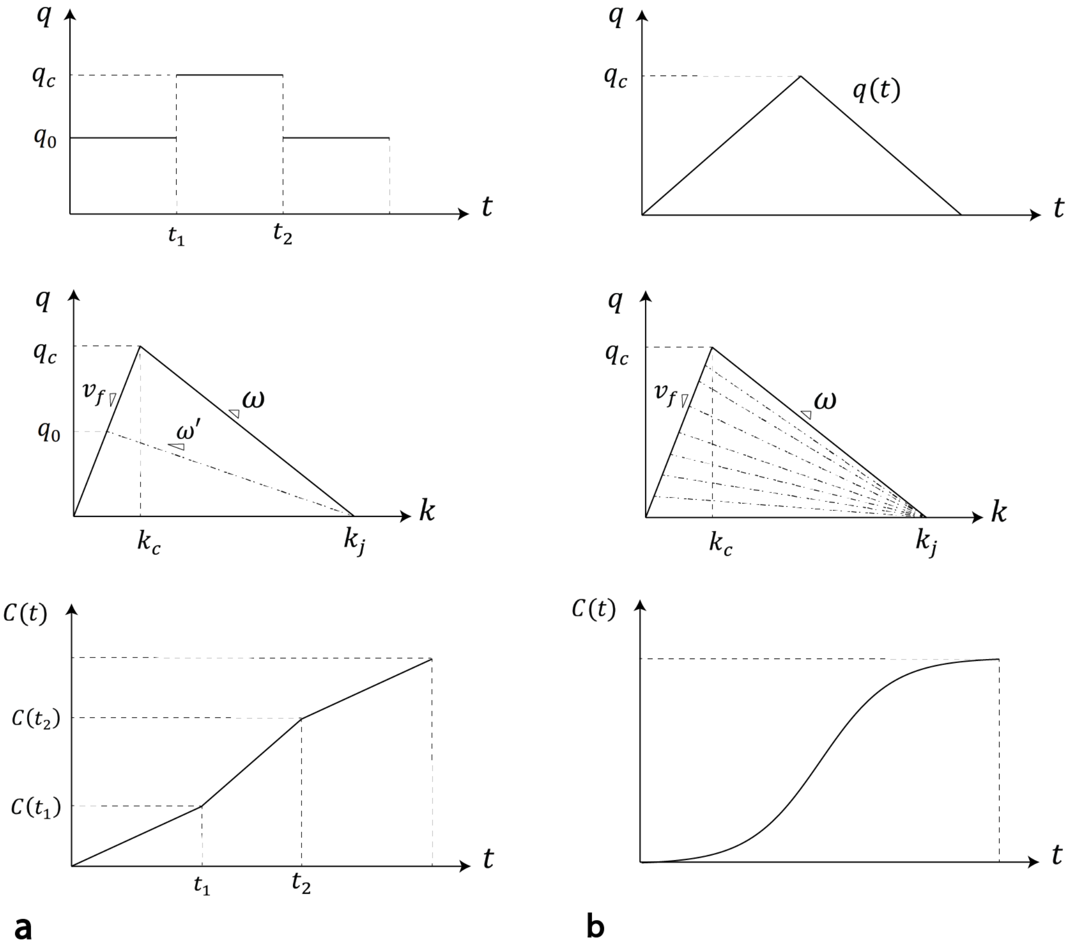

Supplementary Figure 2. Extension of the single corridor to a case with (a) time-dependent step-wise demand profile leading to a piece-wise $C(t)$ and (b) continuous demand profile $q(t)$ leading to a s-shaped continuous $C(t)$.

Supplementary Note 2. Relaxing the homogenous mixing assumption

Here, we extend the SIR model used in the main article, by relaxing the homogenous mixing assumption (Moreno et al., 2002). We use the same directed network structure studied in the main text with *N* links, in which congestion propagates starting from a set of initially congested links. Network nodes have non-uniform distribution of in/out-degrees which introduces heterogeneity to the number of contacts in population of links. In effect, each link ending at node *i* is in contact at its downstream with the number of links equal to node *i*'s out-degree. The street network structure is represented by an adjacency matrix *A* in which each a_{ij} element is equal to 1 if there is a link from node *i* to node *j*, otherwise it is equal to 0. For a link connecting node *i* to node *j*, the actual number of effective contacts is the link's connectivity, denoted here by *k'*, calculated by the sum of the *j*-th row in matrix *A*; i.e. out-degree of node *j*. To incorporate the heterogeneity of effective contacts implied by the network structure, into the SIR model, we define the density of congested $c_{k'}(t)$, free flow $f_{k'}(t)$, and recovered links $r_{k'}(t)$ for different classes of links separated according to their connectivity *k'*. Within each class of links, the normalization condition remains intact and we have

$$c_{k'}(t) + f_{k'}(t) + r_{k'}(t) = 1 \tag{6}$$

and the model described by the system of ordinary differential equations (ODEs) in Eqs. 3-5 (in the main text), is modified as below, to explain the dynamics separately for each connectivity class of links in the network

$$\frac{dc_{k'}(t)}{dt} = -\mu c_{k'}(t) + \beta k\, \theta(t)\big(1 - r_{k'}(t) - c_{k'}(t)\big) \tag{7}$$

$$\frac{dr_{k'}(t)}{dt} = \mu c_{k'}(t) \tag{8}$$

$$\frac{df_{k'}(t)}{dt} = -\beta k\, \theta(t)\big(1 - r_{k'}(t) - c_{k'}(t)\big) \tag{9}$$

where β represents the congestion propagation rate, and μ represents the congestion recovery rate in the network. The function $\theta(t)$ describes the temporal probability of being in contact with a congested link for any randomly selected link on the network, and it can be written as

$$\theta(t) = \frac{\sum_{k'} k' P(k') c_{k'}(t)}{\sum_{k'} k' P(k')} \tag{10}$$

where $P(k')$is the probability that a randomly drawn link from the network is from the connectivity class k'. We estimate the parameters β and μ for the formulated system of ODEs in Supplementary Equations. 7-9 similar to what was previously described in section S3 and in the main manuscript (*Methods*). Supplementary Figure 3 illustrates the estimated β and μ for different values of ρ when homogenous mixing assumption is relaxed.

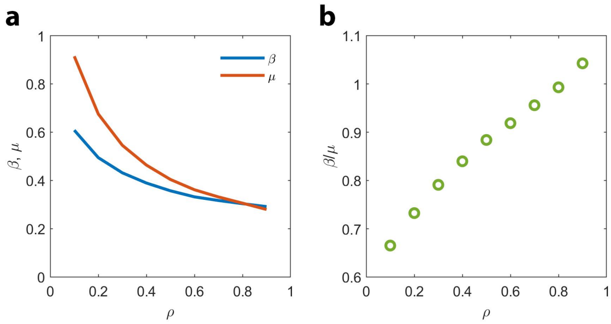

Supplementary Figure 3. (a) The estimated parameters β and μ for the Melbourne's road network for different values of ρ. (b) The ratio between congestion propagation and dissipation rates as a function of ρ, on the network with incorporated heterogeneity of contact.

Supplementary Note 3. Simulation-based dynamic traffic assignment model of Melbourne

Melbourne network consists of 2,974 traffic zones, 55,719 links, and 24,502 nodes. See Supplementary Figure 4. The geometrical configuration of the network is obtained from the Victoria Integrated Transport Model (VITM). Links in the network have multiple physical attributes including number of lanes, capacity, and free flow speed. The link traffic flow fundamental diagrams are calibrated using empirical data from hundreds of loop detectors across the entire network (Gu et al., 2017). Nodes include two major attributes of permitted turning movements and signal control parameters. The network model also includes 1,504 actuated signal controls performing based on historical SCATS data containing information on the cycle time, the turning movements associated with each signal phase, and the minimum green times. The maximum cycle time varies between 60 and 180 seconds across the network. Since most actuated signals are located at major intersections, the maximum cycle time is set at 180 seconds. Once a phase becomes active, the minimum green time is assigned which can be extended to the maximum value depending on the real-time vehicular demand. If no vehicle is detected in an approach, the corresponding signal phase will be skipped. Static origin-destination travel demand data is obtained from the VITM for the four-hour morning peak period (6:00 to 10:00 AM). This includes a total of 2,173,306 equivalent passenger car units. A time-dependent origin-destination demand is estimated for the selected period with 15-minute time intervals using a bi-level optimization approach and empirical traffic counts across the network (Shafiei et al., 2018).

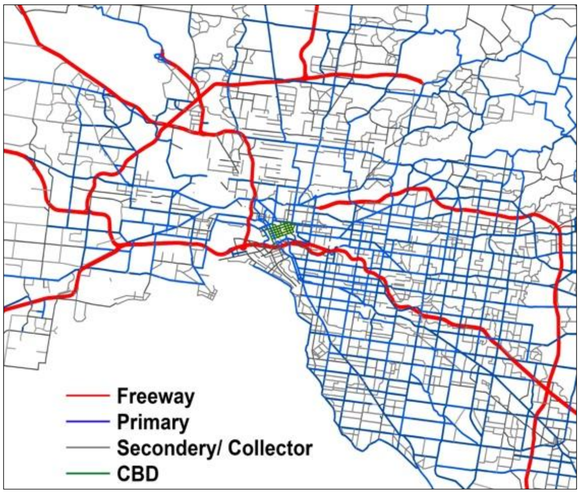

Supplementary Figure 4. Large-scale Melbourne metropolitan area network. Figure from Shafiei et al., 2018.

Supplementary Note 4. Comparing empirical and simulation-based data from the Melbourne network

Here, we provide a comparison of the congestion patterns obtained from the simulation-based dynamic traffic assignment model of Melbourne and Google data from Melbourne. We plot the estimated ratio of β over μ (See Supplementary Figure 5(a)) and evolution of $c(t)$ over time (See Supplementary Figure 5(b)) for both the simulation model and empirical data. The observed and simulated patterns are consistent. However, a disparity is identified with regard to ρ. As explained in the main manuscript, smaller ρ values imply that congestion on links is more likely to spill back onto upstream links; higher ρ values, in contrast, result in uncongested or mildly congested links. Therefore, we argue that smaller ρ values are more appropriate to be used as they better reflect congestion spreading pattern.

However, we believe that the difference observed in the simulation model versus empirical data in smaller ρ values is the effect of the black-box process and speed normalization performed by Google. The distribution of the individual link speeds, obtained from Google, in all cities follows a surprising well-shaped lognormal distribution. See Supplementary Figure 6(a). However, in the simulation model, we observe a considerably larger number of links with free flow speed as what one would expect in real

life. Whereas, Google's speed anomaly might have something to do with its navigation related applications (e.g. providing approximate time of journey). To further analyze the difference between empirical data and our simulation model, we have also plotted the distribution of individual link speed differences (kmph) as shown in Supplementary Figure 6(b). Results suggest that although the simulated traffic data do not exactly follow the empirical data provided by Google at the microscopic level, as expected, the difference between the simulated and empirical data is not significant and provides a solid basis for a comparative analysis at the macroscopic level.

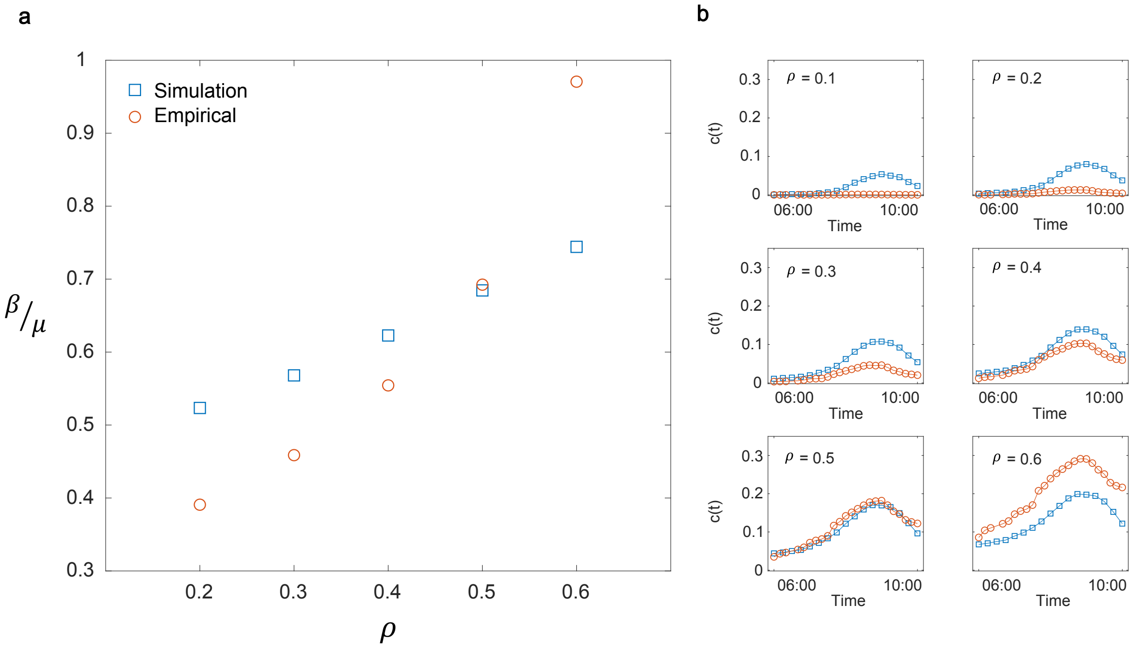

Supplementary Figure 5. (a) Comparison of estimated β/μ in Melbourne obtained from simulation and empirical data in relation to ρ; (b) comparison of evolution of fraction of congested links in the Melbourne network over time for different values of ρ.

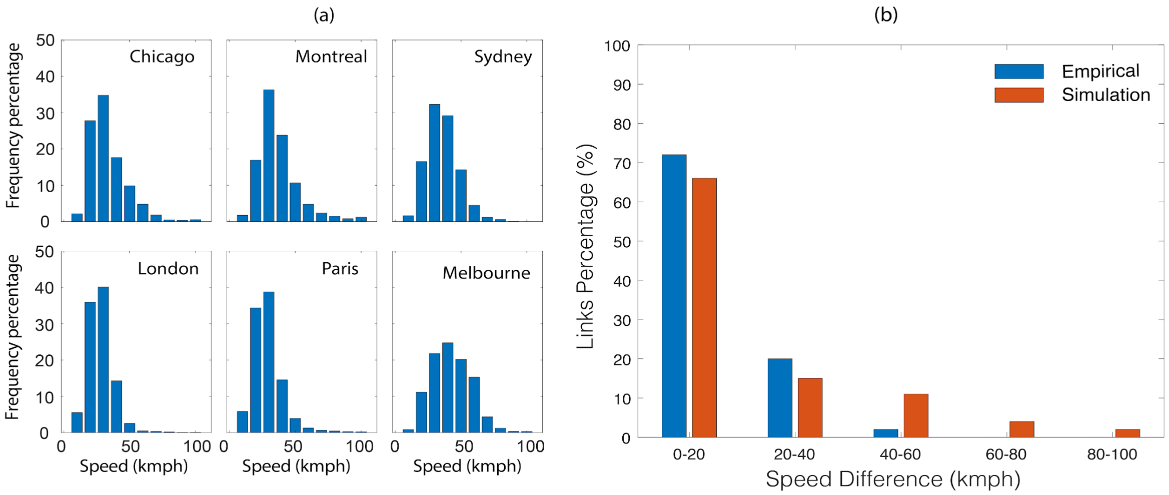

Supplementary Figure 6. (a) Histograms of individual link speeds at 7:00 AM for the studied cities, where x-axis represents the speed in kmph and y-axis is the percentage of links in the network; (b) Difference in link speeds during the morning peak 6:00-10:00 AM, x-axis represents the individual link speed difference in kmph and y-axis is the percentage of links in the network.

Here, we also provide snapshots of the network details in the simulation model vs. empirical data. In the simulation model, all freeway links are composed of several smaller segments and hence, localized congestion on freeways are better captured. The Google traffic data that are used also consider several

segments on long freeways/corridors. Supplementary Figure 7 shows examples of snapshots of a few freeway links from the simulation model vs. Google point observations of speed (mid-link).

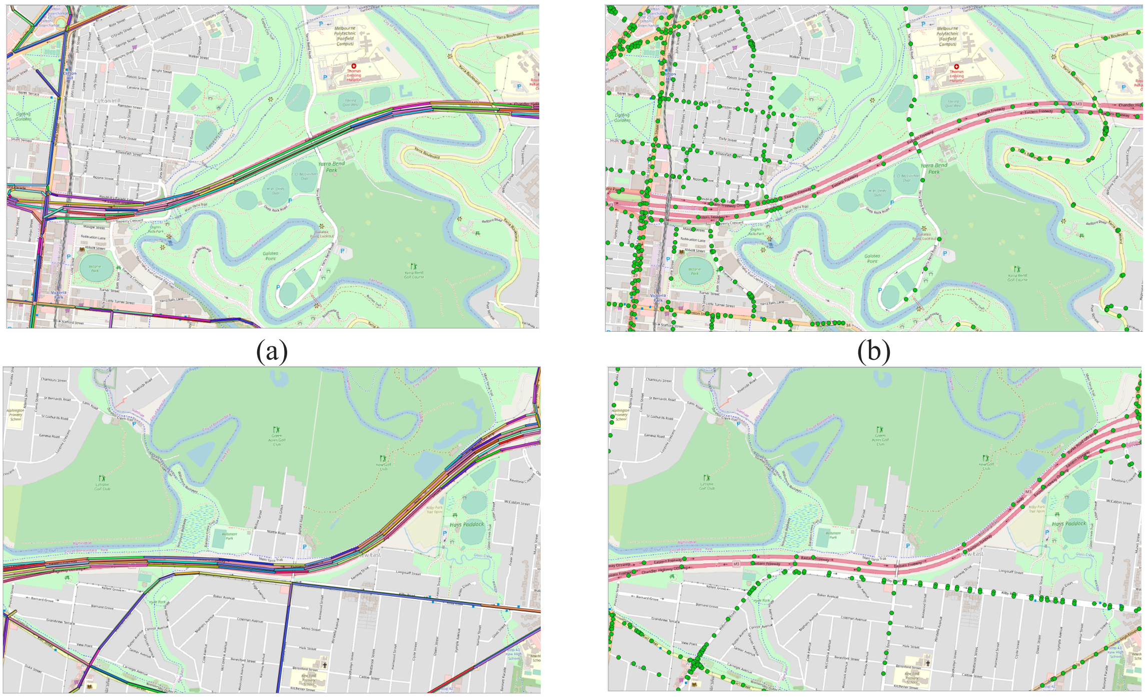

Supplementary Figure 7. Snapshot of the network details and link composition in two different locations in (a and c) the simulation-based dynamic traffic assignment model of Melbourne and (b and d) the empirical data obtained from Google.

Supplementary Note 5. Parameter estimation with pattern search polling

To estimate the parameters of the SIR model, we follow a similar approach to Marinov et al. (2014) in which we formulate the problem as a minimization. The estimation problem seeks to minimize the root mean square error (RMSE) of the predicted fraction of congested links $c(t)$ versus observed fraction of congested links $\hat{c}(t)$. The optimization is solved using the pattern search algorithm available in MATLAB (Audet and Dennis, 2003).

Supplementary Table 1. Estimated model parameters β and μ and associated RMSE for different values of ρ for 4-hour simulation results of the Melbourne network.

ρ	β	μ	RMSE
0.1	0.4033	0.8125	0.0170
0.2	0.2656	0.5156	0.0306
0.3	0.0781	0.1250	0.0407
0.4	0.0289	0.037	0.0164
0.5	0.0233	0.0284	0.0220
0.6	0.0193	0.0229	0.0276
0.7	0.0165	0.0193	0.0320
0.8	0.0146	0.0169	0.0361
0.9	0.0139	0.0154	0.0399

Supplementary Note 6. Potential traffic control applications

The proposed SIR model can be used for adaptive and predictive control of congestion propagation in a network. It can practically be implemented by monitoring the network in real-time and quantifying the number of congested links. The modeling framework can be used to identify time-optimal control strategies with different objectives such as minimizing the total duration of congestion, minimizing the total number of congested links, or minimizing recovery time representing the time that the fraction of congested links in the network reaches a threshold. Similar to Macroscopic Fundamental Diagram (MFD) or Network Fundamental Diagram (NFD)-based control applications, we can improve the modeled system by controlling the inputs such that demand for travel in the network is metered or the recovery rate in the system increases by different means such as improved signal timing, bottleneck removal, capacity increase, etc.

Here, we only conceptually explore a control objective as an example such that the full or partial recovery time of congestion is minimized, given a fixed positive recovery threshold ε. Recovery time T is defined as the first time that the fraction of congested links in the network reduces to ε. For a full recovery $\varepsilon = 0$, while a partial recovery is represented by $0 < \varepsilon < 1$. We consider $u(t)$ as the general control policy rate in which $0 < u < u^{\max}$. Following Bolzini et al. (2017) approach who investigated time-optimal control strategies in epidemic models, we can re-write the SIR dynamics equations in a compact format.

$$x(t) = \begin{pmatrix} c(t) \\ r(t) \end{pmatrix} \text{ and } \dot{x}(t) = f(x(t)) \tag{11}$$

We now add a control term to Supplementary Equation 11.

$$\dot{x}(t) = f(x(t)) + u(t)g(x(t)) \tag{12}$$

where g represents a control policy. A potential control strategy is to reduce the basic reproduction number R_0 by reducing the congestion propagation rate β which can be expressed as

$$g\big(x(t)\big) = \begin{pmatrix} -\beta(k-1)c(t)\big(1 - r(t) - c(t)\big) \\ 0 \end{pmatrix} \tag{13}$$

in which controlled β can be translated to various travel demand management (TDM) strategies. Therefore, a potential optimal control problem can be formulated as

$$\text{Minimize } z(u) = \int_0^T 1dt \tag{14}$$

subject to Supplementary Equation 12 for $t \geq 0$ which represents the congestion propagation model, the boundary condition $x(0) = x_0$ where $x(T) \in \{(c, r): c = \varepsilon\}$, and control rate $0 < u < u^{max}$. Note that $c(0)$ must be strictly greater than ε. Therefore, an optimal control $u^*(t)$ can then be identified, so that the controlled reproduction number is $\beta(k-1)(1 - u^*(\mathrm{t}))/\mu$. For simplicity, one can consider a constant $u^*(t) \equiv u^{max}$ with no delay. Alternatively, one can also consider a delayed control in which $u^*(t)$ changes from 0 to u^{max} at a switching point τ^* which represents the start time of the intervention. If a constant maximum control is assumed, $\tau = 0$. See Supplementary Figure 8 for a hypothetical case in which increasing u reduces the recovery time T given a fixed intervention time $\tau > 0$ and $\varepsilon > 0$. Since the control rate u is a macroscopic control strategy at the network level, translation of the optimal rate u^* to actions at the operations level requires further research in which multiple operational control strategies such as perimeter control, pricing, mode shift, departure time shift can be explored.

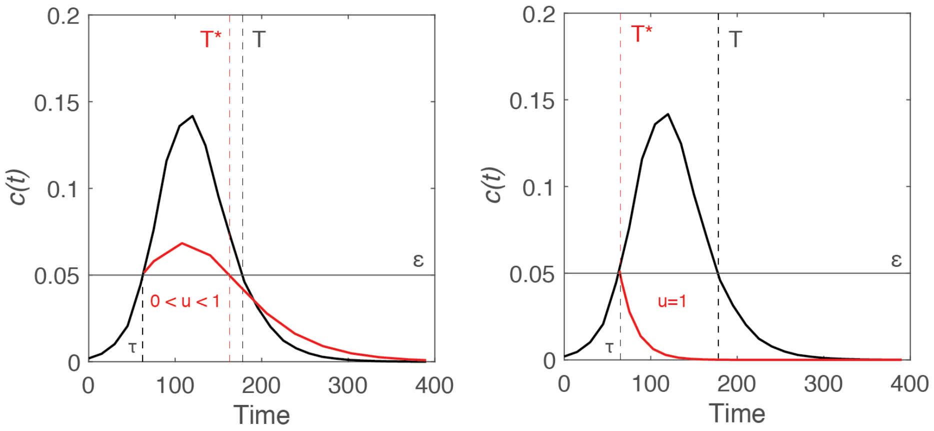

Supplementary Figure 8. Schematic illustration of controlled system (red curve) versus the uncontrolled system (black curve) when $0 < u < 1$ and $u = 0$ in hypothetical case in which the recovery threshold $\varepsilon = 0.05$. Increasing the control rate u, decreases the recovery time T to T^*.

Supplementary Note 7. Network size implications: Sioux Falls case

The presented SIR-based model has been shown to work on large-scale networks, which corresponds to the objective of proposing a macroscopic congestion model that is applicable to real-world networks of actual sizes. See Supplementary Table 2. Given the proposed model is parsimonious and macroscopic, we believe the size of the network, no matter how large, does not affect the model applicability. As long as congestion forms and propagates in a network, the model is expected to reasonably predict the evolution of fraction of congested links over time.

Supplementary Table 2. A summary of the size of the studied networks.

City	Size of the network (number of links)
Paris	121,993
London	144,354
Chicago	102,641
Montreal	49,643
Sydney	33,790
Melbourne	82,857
Melbourne (simulation model)	34,910

For very small networks (e.g. Sioux Falls), in which congestion may quickly propagate to the entire network in a short time span, the model also provides reasonable predictions. Here, we apply the model to describe the propagation of congestion in the Sioux Falls network and show that the model does indeed provide as good predictions as in the other larger scale networks. Supplementary Figure 9(a) illustrates the evolution of fraction of congested links c(t) over time for different values of ρ confirming the previously observed patterns for the propagation and dissipation of congestion in other networks. Supplementary Figure 9(b) shows the SIR model fitting results for $\rho = 0.3$ as an example. When ρ increases, the quality of the estimation is not as good as anticipated mainly due to the small number of links in the network and how quickly a large fraction of the network can become congested in a short time period. Supplementary Figure 9(c) shows the relationship between ρ and R_0 which is consistent with what was observed in other larger scale networks. However, the values of R_0 are relatively large mainly due to the small size of the network which indicates a very fast spread of congestion across the network. The layout and size of the Sioux Falls network is illustrated in Supplementary Figure 10.

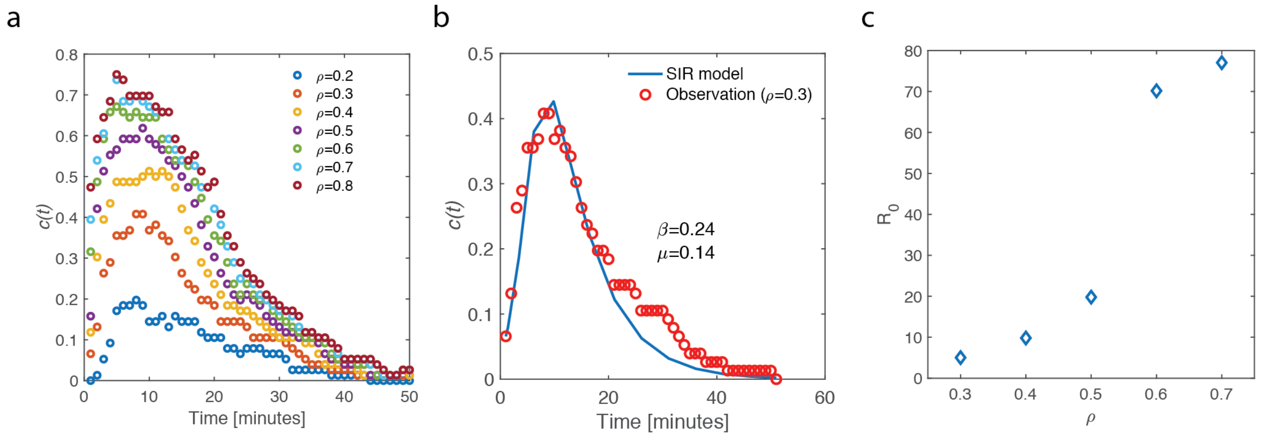

Supplementary Figure 9. (a) Evolution of $c(t)$ over time for different values of ρ in the Sioux Falls network; (b) Comparative illustration of the observed $c(t)$ curve and the predicted $c(t)$ curve by the proposed SIR model when $\rho = 0.3$; (c) Relationship between R_0 and ρ in the Sioux Falls network.

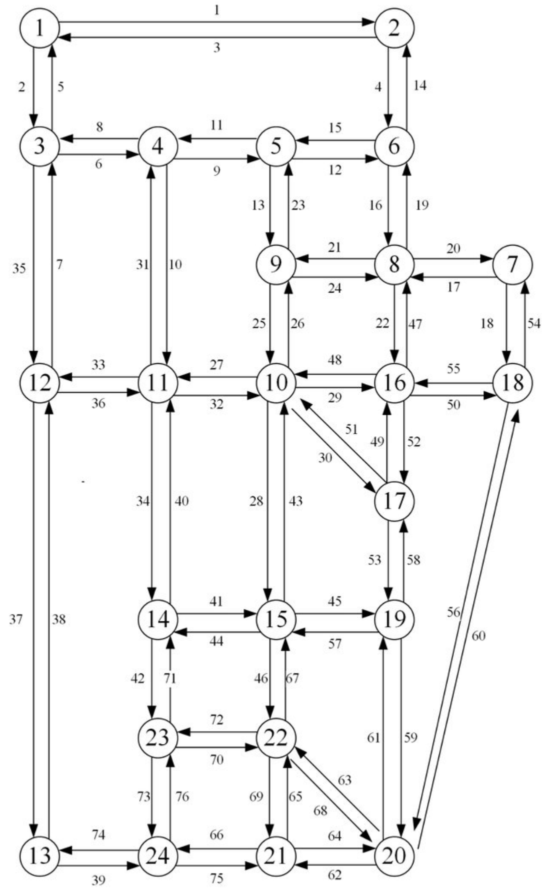

Supplementary Figure 10. Sioux Falls network layout.

Supplementary References

1. Audet, C. & Dennis, J.E. Analysis of generalized pattern searches. *SIAM J. Optim.* **13**, 889-903 (2003).
2. Bolzini, L., Bonacini, E., Soresina, C. & Groppi, M. Time-optimal control strategies in SIR epidemic models. *Math. Biosci.* **292**, 86-96 (2017).
3. Gu, Z., Saberi, M. & Sarvi, M. A big data approach for clustering and calibration of link fundamental diagrams for large-scale network simulation applications. *Transp. Res. C Emerg. Technol.* **94**, 151-171 (2018).
4. Shafiei, S., Gu, Z. & Saberi, M. Calibration and validation of a simulation-based dynamic traffic assignment model for a large-scale congested network. *Simul. Modell. Pract. Theory* **86**, 169-186 (2018).
5. Marinov, T.T., Marinova, R.S., Omojola, J. & Jackson, M. Inverse problem for coefficient identification in SIR epidemic models. *Comput. Math. Appl.* **67**, 2218-2227 (2014).
6. Moreno, Y., Pastor-Satorras, R. & Vespignani, A. Epidemic outbreaks in complex heterogeneous networks. *Eur. Phys. J. B* **26**, 521-529 (2002).
